# Phosphocreatine Attenuates Isoproterenol-Induced Cardiac Fibrosis and Cardiomyocyte Apoptosis

**DOI:** 10.1155/2019/5408289

**Published:** 2019-01-08

**Authors:** Hui Dai, Liang Chen, Dongyue Gao, Aihua Fei

**Affiliations:** Department of Emergency, Xinhua Hospital Affiliated to Shanghai Jiao Tong University School of Medicine, Shanghai, China

## Abstract

The present study was designed to further explore the role and the underlying molecular mechanism of phosphocreatine (PCr) for cardiac fibrosis* in vivo*. Isoproterenol (ISO) was used to induce cardiac fibrosis in rats. PCr administration ameliorated fibrosis by reducing collagen accumulation and fibrosis-related signals, including transforming growth factor beta 1 (TGF-*β*1), alpha smooth muscle actin (*α*-SMA), collagen type I, and collagen type III. Mitogen-activated protein kinases (MAPKs) and nuclear factor kappa B (NF-*κ*B) signaling pathways, including p38, extracellular signal regulated kinase (ERK), c-Jun N-terminal kinase (JNK), and p65, were highly activated by ISO and blocked by PCr. Moreover, PCr decreased ISO-induced matrix metalloproteinase-9 (MMP-9) and increased the tissue inhibitor of metalloproteinase-1 (TIMP-1) expression. Furthermore, PCr suppressed cardiomyocyte apoptosis induced by ISO, as shown by downregulated expression of the proapoptotic caspase-3, Bax, and upregulated expression of the antiapoptotic Bcl-2. Taken together, PCr can be an effective agent for preventing cardiac fibrosis and cardiomyocyte apoptosis.

## 1. Introduction

Cardiac fibrosis is a common pathological change in various heart diseases that develops to a certain stage and is characterized by excessive extracellular matrix (ECM) deposition [1]. Cardiac fibrosis can result in diastolic dysfunction and arrhythmia, ultimately leading to heart failure [2]. The development of fibrosis depends on upregulation of MMPs and downregulation of TIMPs, activation of profibrotic mediators, differentiation of fibroblasts into myofibroblasts, and endothelial-to-mesenchymal transition (EndMT) [3–5]. Cardiac fibroblasts, the most abundant cell type in the myocardium, play a critical role in the process of fibrosis. Many mediators induce the transformation of fibroblasts into myofibroblasts, which is characterized by expression of *α*-SMA, proliferation, migration, and release of proinflammatory signals, and increased production of ECM remodeling proteins. Inflammatory factors, cytokines, and signaling pathways can interact with one another to form a complex network to promote the continuous development of cardiac fibrosis. A growing body of evidence has focused on slowing, preventing, or even reversing the progress of cardiac fibrosis; unfortunately, there have been few effective therapies to date.

Apoptosis is a process of intrinsic cell death caused by various factors, which is attributed to programmed cell death. Apoptosis is critical for embryonic development, homeostasis, and cancer. Cardiomyocyte apoptosis was first reported in the mid-1990s to explain acute ischemic death of cardiomyocytes in rabbits and in humans with acute myocardial infarction [6, 7]. Since then, most studies have shown that cardiomyocyte apoptosis plays an important role in the progression of heart failure in almost all types of heart disease [8]. There is a significant association between improved cardiac function and prevention of cardiomyocyte apoptosis.

PCr is an important substance involved in the energy metabolism and is an important energy supply source for cells and adenosine triphosphate [9]. A large number of studies have confirmed that PCr can improve cardiac energy metabolism, preserve cardiac excitability, conductivity, and contractility, reduce the incidence of arrhythmia, and protect cardiac function [10]. As a myocardial protector, PCr is now widely used for extracorporeal circulation, coronary heart disease, heart failure, and myocardial infarction [11, 12].

In the previous study, we demonstrated that PCr suppressed cardiac fibrosis in rat cardiomyocytes through modulation of MAPK and NF-*κ*B pathways [13]. In this study, we further verify the effect of PCr* in vivo* on cardiac fibrosis and cardiomyocyte apoptosis in ISO-induced rat model.

## 2. Methods

### 2.1. Reagents

ISO was purchased from Sigma-Aldrich (St. Louis, MO, USA) and dissolved in phosphate-buffered saline (PBS). PCr sodium was purchased from Sangon Biotech (Shanghai, China).

### 2.2. Animals and Treatment

Male Sprague Dawley rats (weighing 180-200 g and aged 8 weeks) from the Shanghai Experimental Animal Center (Shanghai, China) were used. All experimental procedures were in accordance with the Guide for the Care and Use of Laboratory Animals and approved by the Animal Care and Use Committee of Xinhua Hospital Affiliated to Shanghai Jiao Tong University School of Medicine. Ninety rats were randomly divided into 3 groups: control, ISO, and ISO+PCr. The control group was treated with saline solution in the process of the trial. The ISO rat model was induced by subcutaneous ISO injection twice a day (50 mg/kg/d for 14 days), followed by intraperitoneal injection daily of PCr (200 mg/kg/d for 45 days) for the ISO+PCr group. The ISO group was given an equal dose of saline solution during the period of administration of PCr in the ISO+PCr group.

### 2.3. Echocardiography

Rats were anaesthetized with 2% isoflurane, and cardiac function was assessed using transthoracic echocardiography (Philips CX50 ultrasound systems). M-mode and two-dimensional echocardiography were performed to assess cardiac parameters, including left ventricular end-diastolic dimension (LVDd), left ventricular end-systolic dimension (LVDs), left ventricular posterior wall thickness (PWT), inter ventricular septum wall thickness (IVST), ejection fraction (EF), and fractional shortening (FS). The average of 3 consecutive cardiac cycles was used for each measurement.

### 2.4. Picrosirius Red Staining

The left ventricles of 8 rats from each group were randomly selected, and euthanasia was performed by cervical dislocation under deep anesthesia with isoflurane after the treatment. The heart tissue specimen was fixed by paraformaldehyde, paraffin embedded, and sectioned at 4 *μ*m thickness. Image-Pro Plus software (Media Cybernetics, Rockville, MD, USA) was used to measure fibrosis from 10 random fields per section. The collagen content of each group was evaluated by the collagen volume fraction (CFV). This was calculated using the following equation: CFV = collagen area/total area ×100%.

### 2.5. Immunohistochemistry

The laminin was stained on 4 *μ*m sections of ventricular tissues. The sections were incubated overnight at 4°C with primary antibody and with peroxidase-conjugated second antibody at 37°C for 20 min. Then the sections were visualized with a DAB-based colorimetric method. The level of laminin was also determined by Image-Pro Plus software.

### 2.6. TUNEL Staining

TUNEL staining was performed using the ApopTag kit (Roche) according to the manufacturer's instructions. The apoptosis content was calculated as apoptotic cells per field.

### 2.7. Western Blotting

The proteins lysed from left ventricle were separated by 10% SDS-polyacrylamide gel and transferred to PVDF membranes using a semidry transferring method. The membranes were sharked for 1 h and incubated with specific primary antibodies at 4°C overnight. The next day, the membranes were incubated in horseradish peroxidase- (HRP-) labeled secondary antibodies (Beyotime, Nanjing, China), colored with fluorescent substrates and captured on the Bio-Rad system. Antibodies against collagen I, collagen III, p-P65, Bcl-2, Bax, CASP-3, GAPDH, and histone H3 were purchased from Abcam (Cambridge, MA, USA). Antibodies against p-ERK, p-JNK, p-P38, TGF-*β*1, *α*-SMA, MMP9, and TIMP-1 were obtained from Cell Signaling Technology (Danvers, MA, USA). GAPDH and histone H3 were used as internal controls.

### 2.8. Statistical Analysis

All results are presented as mean ± SD using GraphPad Prism 7 (GraphPad Software, Inc.). Statistical analyses were performed using SPSS Statistics 20 (IBM) and GraphPad Prism. For comparison between three groups, one-way ANOVA with the Bonferroni for post hoc test was performed.* P*<0.05 was considered statistically significant.

## 3. Results

### 3.1. Effect of PCr on Left Ventricular Dimensions and Systolic Function in the ISO-Induced Rat Model

To assess changes of the cardiac structure and function in response to ISO or/and PCr treatment, echocardiography was performed following treatment. As shown in Table 1, the heart rate (HR), PWT, and IVST were significantly increased in the ISO group (*P*<0.01 versus control group) and were decreased in the ISO+PCr group. The LVDd, LVDs, EF, and FS were lower in the ISO group than those in the control group, and PCr improved the LVDd, LVDs, EF, and FS slightly. These responses were insignificant between ISO and ISO+PCr groups but were significant compared to the control group.

### 3.2. Effect of PCr on ISO-Induced Heart and Left Ventricular Mass

Previous studies showed that ISO can induce the cardiac hypertrophy. We assessed the changes of hearts weight among three groups. The hearts weight (HW) and left ventricular weight (LVW) of ISO group were heavier than that in control group and ISO+PCr group (Table 2). Heart weight to body weight ratio (HW/BW) and left ventricular weight to the body weight ratio (LVW/BW) were lower in the ISO+PCr group than that in the ISO group; however, their results were not statistically different. The body weights of the rats were stable, with no significant differences among the control, ISO, and ISO+PCr groups.

### 3.3. PCr Decreased the ISO-Induced Cardiac Fibrosis in Rat Model

**Figure 1:**
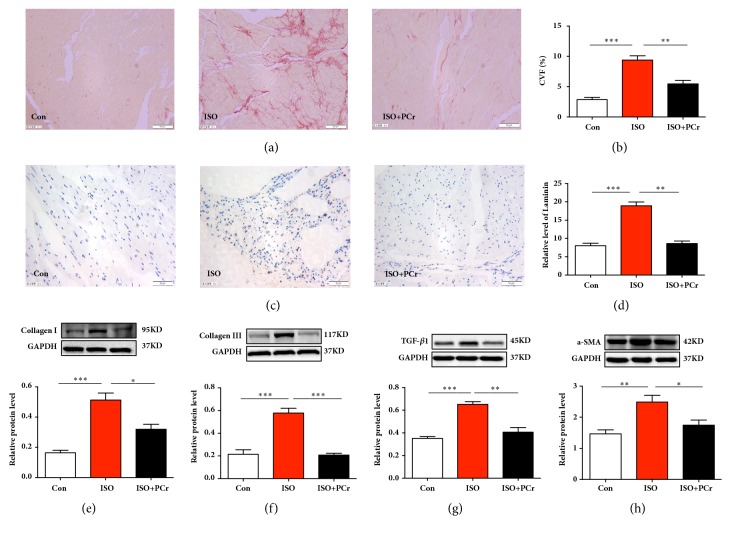
PCr inhibited ISO-induced cardiac fibrosis. (a-b) Picrosirius red staining and the CVF of each group. (c-d) Laminin level between each group. (e–h) Western blots and quantification of collagen I, collagen III, TGF-*β*1, and *α*-SMA protein expression in left ventricular tissues. *∗P<0.05*, *∗∗P<0.01*, and *∗∗∗P<0.001*, versus the ISO group, n=8 per group. Scale bar=50 *μ*m.

We initially detected the collagen and laminin deposition of left ventricular tissues with picrosirius red staining for collagen and immunohistochemistry for laminin (Figures 1(a)–1(d)). Compared with the control group, the collagen deposition was significantly increased in ISO group and decreased with PCr treatment, as well as the laminin results. The protein expression of collagen I, collagen III, and fibrosis-related molecules TGF-*β*1 and *α*-SMA was also evaluated by Western blotting. ISO was found to induce dramatic increases in levels of these proteins, whereas treatment with PCr attenuated these ISO-induced changes (Figures 1(e)–1(h)). These results preliminarily indicated that PCr can prevent ISO-induced cardiac fibrosis to some extent.

### 3.4. PCr Block ISO-Induced MAPK and Pathway Activation

**Figure 2:**
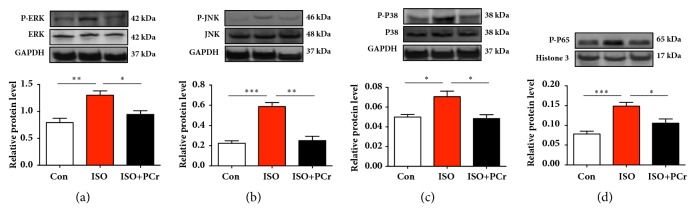
PCr prevented ISO-induced cardiac fibrosis through MAPK and NF-*κ*B pathway. (a–c) The expression of p-ERK, p-JNK, and p-P38 was analyzed by Western blotting. (d) NF-*κ*B in nuclear extraction was analyzed. *∗P<0.05*, *∗∗P<0.01*, and *∗∗∗P<0.001*, versus the ISO group, n=8 per group.

MAPKs and NF-*κ*B signaling pathways have been reported to be important for fibrosis progression in various organs [14, 15]. Here, we also explore if PCr could attenuate fibrosis from preventing MAPKs/NF-*κ*B activation. As shown in Figures 2(a)–2(c), Western blotting analysis suggested that ISO triggered overexpression of phosphorylated p38, ERK, and JNK. Significantly, PCr reduced these proteins activation in comparison to the ISO group. Further, the PCr can decrease NF-*κ*B activation induced by ISO by inhibiting nuclear extraction (Figure 2(d)). Taken together, the data above suggested that PCr prevented ISO-induced fibrosis relied on suppression of MAPKs and NF-*κ*B signaling pathways.

### 3.5. PCr Downregluated the MMP-9 and Upregluated the TIMP-1 Expression

**Figure 3:**
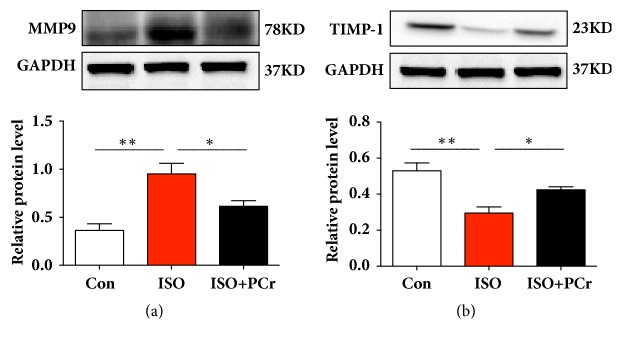
PCr regulated the expression of MMP-9 and TIMP-1. (a) Western blot of protein expression of MMP-9. (b) Expression of TIMP-1 protein. *∗*P<0.05; *∗∗*P<0.01, versus the ISO group, n=8 per group.

One of the causes of cardiac fibrosis is the increased MMP expression and the reduced TIMP expression. MMP-9 can cleave ECM protein and plays an important role in cardiac fibrosis [16]. We assessed the change of MMP-9 and TIMP-1 in the hearts of model rat. The expression of MMP-9 was significantly increased in ISO group compared with that in the control group but decreased with PCr treatment (Figure 3(a)). In contrast, the TIMP-1 expression was dramatically decreased with the ISO-induced cardiac fibrosis compared to the control group. With the PCr treatment, the expression was significantly increased (Figure 3(b)).

### 3.6. PCr Reduced ISO-Induced Cardiomyocyte Apoptosis in the Rat Model

**Figure 4:**
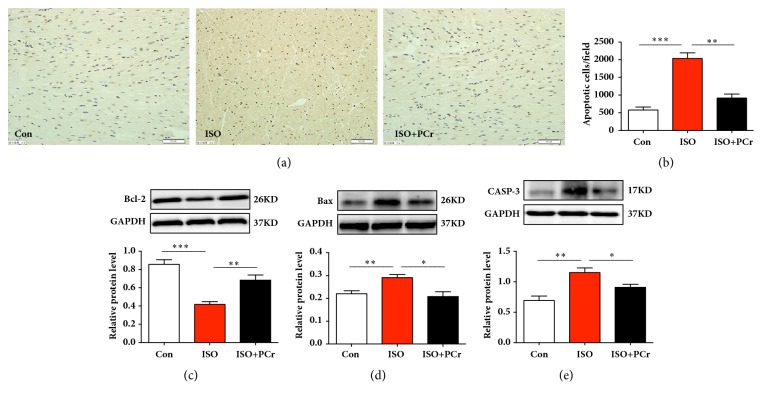
PCr decreased cardiomyocyte apoptosis. (a) Results of TUNEL staining. (b) The number of apoptotic cells per field. (c–e) Western blots of Bcl-2, Bax, and CASP-3 protein. *∗*P<0.05, *∗∗*P<0.01, and *∗∗∗*P<0.001, versus the ISO group, n=8 per group. Scale bar=50 *μ*m.

In addition to cardiac fibrosis, we further studied the role of PCr on ISO-induced cardiomyocyte apoptosis in the rat model. Firstly, we used the TUNEL staining to detect the apoptotic cardiomyocyte in the Con, ISO, ISO+PCr groups, respectively. The apoptotic cardiomyocyte was decreased with the PCr treatment, compared with the ISO treatment (Figures 4(a) and 4(b)). Next, we verified the changes of antiapoptotic Bcl-2 protein and proapoptotic Bax and CASP-3 protein by Western blot (Figures 4(c)–4(e)). With the ISO administration, the Bcl-2 protein level was decreased, whereas Bax and CASP-3 protein levels were increased in myocardial tissue compared with the control group. PCr treatment in ISO administered rats reduced the apoptosis by raising the level of Bcl-2 protein and lowering the levels of Bax and CASP-3 protein. Furthermore, ISO increased the apoptosis of myocardium cells, while the apoptotic cells were sharply reduced in the PCr group. Treatment with PCr was also helpful for ameliorating apoptosis in ISO administered rats.

## 4. Discussion

The results of the present study demonstrated that PCr significantly ameliorated the aberrant interstitial collagen deposition in ISO-induced rat hearts. The cardioprotective role of PCr was attributed to its inhibitory effect on the cardiomyocyte apoptosis and cardiac fibrosis.

Animal models have long been used to explore the pathogenesis and molecular mechanisms of cardiovascular diseases to improve the diagnosis, prevention and treatment of cardiac disease [17]. Many different animal models were used to study cardiac fibrosis, such as spontaneously hypertensive induced model [18], the surgical induced models, and drug induced models [19–21]. The ISO-induced model has been widely used to induce cardiac hypertrophy, fibroblast activation, and cardiac fibrosis [22–26], which is similar to disease procession, relatively noninvasive and low animal mortality. High doses of ISO cause cardiac energy depletion, which leads to a series of biochemical and structural changes in cardiomyocytes [27]. Mechanisms of oxidative stress, inflammation activation, MMP activation, MAPKs pathway, and cardiomyocyte apoptosis have been reported to be associated with ISO-induced cardiac fibrosis [28–31]. However, the way of administration and dose of ISO reported in the literature vary widely, which have different contributions to the survival of cardiomyocytes. In our study, adult male rats were treated with ISO (50 mg/kg/d) for two weeks to establish the model of cardiac fibrosis. Throughout the experiment, one, five, and three rats died in the control, ISO, and ISO+PCr groups, respectively. The mortality of the three groups did not affect subsequent experiments. After 14 days of ISO treatment, we found that collagen, *α*-SMA, and apoptosis were significantly increased in rat hearts tissue. These results fully support the fact that our modeling is feasible. After PCr treatment, the markers of cardiac fibrosis and cardiomyocyte apoptosis can be greatly improved. However, PCr did not significantly improve the structure and function of the heart, such that LVDd, LVDs, IVST, PWT, and EF values have not been significantly changed between ISO and ISO+PCr groups. We hypothesized that these macrodata changes may require PCr to administrate longer period.

Studies have shown that the MAPK signaling pathway is involved in organ fibrosis, through regulating cellular response to growth, apoptosis, and stress signals [32–35]. Tsukada et al. have shown that MAPK not only increases the expression of collagen I, but also increases the stability of collagen I mRNA induced by TGF-*β* treatment [34]. Activation of the MAPK signaling pathway can further activate NF-*κ*B, leading to nuclear translocation of NF-*κ*B for target gene transcription activation. Wang et al. have demonstrated that NF-*κ*B is a crucial factor in fibroblast growth and collagen expression mediated by Ang II and tumor necrosis factor alpha (TNF-*α*) [36]. Our results suggest that PCr can reverse the development of cardiac fibrosis by inhibiting the activation of the MAPK and NF-*κ*B pathways to a certain degree.

Increased synthesis of MMPs and downregulation of TIMPs by molecular signals, such as NF-*κ*B, promote the development of cardiac fibrosis. MMP-9 is a zinc-containing endoprotease known to cleave matrix substrates, such as collagen type I and collagen type IV. The expression of MMP-9 was found to be upregulated in cardiac fibrosis. Using the MMP-9 knockout rat model, Ducharme et al. confirmed that knockout of MMP-9 in myocardial infarction preserved some of the cardiac function and reduced cardiac fibrosis [37]. In the present study, PCr treatment significantly attenuated the MMP-9 synthesis and activities induced by ISO. In addition, the TIMP-1 level was found to considerably decrease in the ISO-induced rat heart in our study; in contrast, PCr markedly elevated the expression of TIMP-1. Therefore, on the other hand, we found that PCr could alleviate the progression of cardiac fibrosis by downregulating the MMP-9 and upregulating the TIMP-1 expression.

Cardiomyocyte apoptosis has been shown to be a critical form of cell death. A key component of the apoptotic signaling pathway is the activation of caspase. Caspase-3 is the most critical apoptosis-regulating protein in the downstream of the caspase family, which is activated by various apoptotic stimuli. In normal organisms, caspase-3 is in an inactive state. When the body undergoes apoptotic stress, caspase-3 can be cleaved and activated by the caspase cascade, which ultimately induces apoptosis [38]. Bcl-2 family proteins have important regulatory functions in the process of apoptosis. Both Bcl-2 and Bax belong to the Bcl-2 family, which have antiapoptotic and proapoptotic effects, respectively [39]. Here, we find that PCr attenuated ISO-induced cardiomyocyte apoptosis by downregulating proapoptotic caspase-3, Bax expression, and antiapoptotic Bcl-2 expression.

In summary, PCr can attenuate ISO-stimulated cardiac fibrosis by inhibiting the MAPK and NF-*κ*B pathways and regulating the expression of MMP-9 and TIMP-1, also inhibiting the ISO-induced cardiomyocyte apoptosis. These findings suggest that PCr can be a potential drug against cardiac fibrosis and cardiomyocyte apoptosis.

## Figures and Tables

**Table 1:** Effect of PCr on the echocardiographic parameters of ISO-induced rats.

	Control	ISO	PCr
Heart rate, beats/min	416.25±11.90	490.38±14.23^*∗*#^	481.88±11.62^*∗*#^
LVDd, mm	6.38±0.26	5.91±0.22^*∗*#^	6.06±0.20^*∗*^
LVDs, mm	3.33±0.18	2.30±0.15^*∗*#^	2.39±0.20^*∗*#^
IVST, mm	1.34±0.07	2.30±0.13^*∗*#^	2.20±0.11^*∗*#^
PWT, mm	1.21±0.10	1.55±0.09^*∗*#^	1.48±0.10^*∗*#^
EF, %	79.63±1.30	73.13±2.36^*∗*#^	74.50±3.42^*∗*#^
FS, %	42.88±2.03	33.75±2.12^*∗*#^	34.75±2.66^*∗*#^

The data are given as the mean ± SD. LVDd, left ventricular end-diastolic dimension; LVDs, left ventricular end-systolic dimension; PWT, left ventricular posterior wall thickness; IVST, inter ventricular septum wall thickness; EF, left ventricular ejection fraction; FS, fractional shortening. The data represent the mean ± SD. ^*∗*^: P < 0.05 versus control group. ^#^: P < 0.01 versus control group, n=8 per group.

**Table 2:** Comparison of heart and left ventricular mass among the groups.

	Con	ISO	PCr
BW (g)	193.6±9.41	188.7±10.59	191.5±7.88
HW (g)	1.12±0.10	1.36±0.06^*∗*#^	1.31±0.07^*∗*#^
HW/BW (mg/g)	5.79±0.57	7.26±0.62^*∗*#^	6.85±0.44^*∗*#^
LVW (g)	0.63±0.09	0.89±0.07^*∗*#^	0.85±0.09^*∗*#^
LVW/BW (mg/g)	3.23±0.42	4.70±0.44^*∗*#^	4.43±0.53^*∗*#^

The data are given as the mean ± SD. BW, body weight; HW, heart weight; HW/BW, ratio of the heart weight to the body weight; LVW, left ventricular weight; LVW/BW, ratio of the left ventricular weight to the body weight. ^*∗*^: P < 0.05 versus control group. ^#^: P < 0.01 versus control group, n=8 per group.

## Data Availability

The data used to support the findings of this study are available from the corresponding author upon request.
